# Inter-individual Relationships between Sympathetic Arterial Baroreflex Function and Cerebral Perfusion Control in Healthy Males

**DOI:** 10.3389/fnins.2017.00457

**Published:** 2017-08-15

**Authors:** Trevor Witter, Yu-Chieh Tzeng, Terry O'Donnell, Jessica Kusel, Bridget Walker, Mary Berry, Chloe E. Taylor

**Affiliations:** ^1^Wellington Medical Technology Group, Centre for Translational Physiology, University of Otago Wellington, New Zealand; ^2^School of Science and Health, Western Sydney University Sydney, NSW, Australia; ^3^School of Medicine, Western Sydney University Sydney, NSW, Australia

**Keywords:** baroreflex sensitivity, cerebral autoregulation, microneurography, muscle sympathetic nerve activity, modified Oxford method

## Abstract

Maintenance of adequate cerebral perfusion during normal physiological challenges requires integration between cerebral blood flow (CBF) and systemic blood pressure control mechanisms. Previous studies have shown that cardiac baroreflex sensitivity (BRS) is inversely related to some measures of cerebral autoregulation. However, interactions between the sympathetic arterial baroreflex and cerebral perfusion control mechanisms have not been explored. To determine the nature and magnitude of these interactions we measured R–R interval, blood pressure, CBF velocity, and muscle sympathetic nerve activity (MSNA) in 11 healthy young males. Sympathetic BRS was estimated using modified Oxford method as the relationship between beat-to-beat diastolic blood pressure (DBP) and MSNA. Integrated control of CBF was quantified using transfer function analysis (TFA) metrics derived during rest and Tieck's autoregulatory index following bilateral thigh cuff deflation. Sympathetic BRS during modified Oxford trials was significantly related to autoregulatory index (*r* = 0.64, *p* = 0.03). Sympathetic BRS during spontaneous baseline was significantly related to transfer function gain (*r* = −0.74, *p* = 0.01). A more negative value for sympathetic BRS indicates more effective arterial baroreflex regulation, and a lower transfer function gain reflects greater cerebral autoregulation. Therefore, these findings indicate that males with attenuated CBF regulation have greater sympathetic BRS (and vice versa), consistent with compensatory interactions between blood pressure and cerebral perfusion control mechanisms.

## Introduction

The dynamic regulation of cerebral blood flow (CBF) is an integrative process that involves multiple homeostatic mechanisms (Ogoh et al., 2008). In humans and many mammalian species, the arterial baroreflex plays a central role by maintaining relatively stable perfusion pressure gradient across the cerebrovascular bed via vagal control of heart rate, and sympathetic control of heart rate, myocardial contractility, and total peripheral resistance (Parati and Bilo, 2012). Cerebral autoregulation (CA) operates at the level of the end-organ by stabilizing CBF against dynamic changes in blood pressure through myogenic and neurogenic mechanisms (Zhang et al., 2002). It is important to distinguish this from static CA, which refers to the regulation of CBF over more gradual changes in pressure (Paulson et al., 1989). References to CA from this point onwards will be specific to dynamic CA. From a dynamic systems perspective, the operation of the arterial baroreflex and CA in tandem provides a safeguard to ensure that the brain receives a stable blood supply. However, if both of these control systems fail then cerebral perfusion may be compromised. As Faes et al. (2013) reported, reductions in both cardiac baroreflex modulation of blood pressure and cerebral autoregulation occur prior to syncope. Furthermore, in individuals prone to syncope head-up tilt is associated with impaired cerebral autoregulation and an inability to activate the cardiac baroreflex compared to individuals not prone to syncope (Bari et al., 2017).

Several previous studies have evaluated the inter-individual relationships between cardiac baroreflex sensitivity (BRS) and CA within healthy young humans and found that individuals with comparatively poor CA appear to be counterbalanced with relatively efficient cardiac BRS and vice versa (Tzeng et al., 2010; Nasr et al., 2014). Additionally, it has been found that markers of blood pressure variability were positively related to metrics of CA, and inversely related to cardiac BRS (Tzeng et al., 2010). These findings indicate that individuals with low cardiac BRS and high blood pressure variability are not necessarily deficient in their integrated capacity to control CBF because they had high levels of CA (and vice versa). Studies involving alternative approaches, such as information transfer analysis, also indicate that cross-talk exists between cerebrovascular and cardiovascular control systems (Bari et al., 2016, 2017). However, whilst such functional counterbalancing may be a possible feature of regulation, to date no studies have taken the sympathetic baroreflex into account. Mechanical deformation of barosensory vessels not only modulates cardiac parasympathetic outflow, but it also elicits reflex changes in sympathetic vascular tone and both components are crucial to the maintenance of stable systemic blood pressure.

The objective of this study was to determine the functional relationship between the vascular sympathetic baroreflex and CA. With respect to both measures, we employed both spontaneous and provocative methods to provide a more comprehensive assessment of potential relationships. We quantified sympathetic BRS using the pharmacological modified Oxford method, and a spontaneous approach that characterizes the dynamic relationships between spontaneous fluctuations in diastolic blood pressure (DBP) and peroneal muscle sympathetic nerve activity (MSNA). To characterize CA we employed the thigh cuff rapid deflation method (Aaslid et al., 1989) and spontaneous transfer function analysis (TFA) of blood pressure and middle cerebral blood velocity fluctuations. Consistent with our previous investigations relating to cardiac BRS, we hypothesize that sympathetic vascular BRS is reciprocally related to CA in otherwise healthy individuals. Cardiac BRS was also computed for comparison using the modified Oxford method and sequence method.

## Materials and methods

### Subjects

Fourteen healthy young male subjects (mean age = 24.4 years; range 19–29) were recruited from the local community. All subjects were nonsmokers, normotensive (blood pressure <140/90 mmHg) and had no prior evidence of cardiopulmonary disease. Subjects abstained from caffeine and exercise for a minimum of 12 h prior to data collection and were asked to have a light breakfast at least 2 h before the study commencement at 08:00 h. All subjects gave written informed consent in accordance with the Declaration of Helsinki. The protocol was approved by the Central Region Health and Disability Ethics Committee. Individuals with very high (>140/90 mmHg) or very low (<100/60 mmHg) resting blood pressure, previous history of stroke, brain surgery or severe head trauma were excluded, as were obese individuals (Body Mass Index >30).

### Measurements

Continuous recordings were made of blood pressure via finger photoplethysmography (Finometer MIDI, Finapres Medical Systems, Amsterdam, Netherlands), three lead electrocardiogram, right middle cerebral artery blood flow velocity (MCAv; 2-MHz pulsed Doppler ultrasound, ST3 Digital Transcranial Doppler System, Spencer Technologies, Seattle, WA), and partial pressure of end-tidal CO_2_ sampled from a nasal line (gas analyzer model ML206, ADInstruments, Colorado Springs, CO). Recalibration of finger photoplethysmography was performed before each section of the experimental protocol to prevent drifting of the measurement, which was also verified against pneumatic brachial artery blood pressure measurements (Cardioscope II, Pulsecor, Auckland, New Zealand). The transcranial Doppler probe was fixated using custom headgear and positioned to measure blood flow velocity at the M1 segment of the middle cerebral artery via the temporal window at a depth of 50–65 mm. Data were continuously acquired at 1 kHz per channel via an analog-to-digital converter (PowerLab/16SP ML795; ADInstruments, Colorado Springs, CO), interfaced with a computer and stored for offline analysis. Data processing and analysis was performed with custom written software using LabView 11 (National Instruments, Austin, TX).

#### Microneurography

Muscle sympathetic nerve activity (MSNA) recordings were acquired from a muscle nerve fascicle in the right or left peroneal nerve, at the level of the fibular head. Nerve activity was recorded using tungsten microelectrodes with a diameter of 200 μm at the shaft and an uninsulated tip of 1–5 μm. A reference electrode was subcutaneously inserted ~1 cm from the recording electrode. Neural activity was amplified with a gain of 20,000 and fed through a bandpass filter with a bandwidth of 0.3–5.0 kHz via an isolated amplifier (NeuroAmp EX, ADInstruments, Dunedin, New Zealand). Microneurographic recordings were deemed acceptable based on previous criteria (Mark et al., 1985). First, weak electrical impulses (0.02–1 mA, 0.2 s, 1 Hz) conducted through the recording electrode elicited involuntary muscle contraction but not paraesthesia. Next, stretching or tapping of the muscles supplied by the nerve fascicle elicited afferent discharges while stroking of the skin did not. Third, spontaneous, intermittent, pulse synchronous bursts were present in the neurogram and increased in frequency and amplitude during phase 2 and 3 of a Valsalva maneuver (Vallbo et al., 1979).

### Experimental protocol

All studies were performed at the University of Otago in a laboratory with ambient temperature controlled between 22 and 23°C. Subjects were in the supine position, with the leg used for MSNA measurement supported to maintain slight knee flexion. After a 10-min stabilization period, baseline recordings were made for 5 min. Subjects then underwent pharmacological BP manipulation via the modified Oxford method followed by the thigh cuff rapid deflation method. The thigh cuff procedure was the final part of the protocol because the movement of the lower limbs generated by this technique, albeit minimal, was likely to dislodge the microelectrode and jeopardize the stability of the MSNA recording.

### Arterial baroreflex sensitivity

Sympathetic BRS was assessed through the use of both spontaneous recordings and the modified Oxford method (Rudas et al., 1999; Lipman et al., 2003; Tzeng et al., 2009). Spontaneous BRS measurements were taken from a single 5-min recording of baseline data, with the subject lying in the supine position. Oxford BRS was quantified through the average of two Modified Oxford recordings. Both trials consisted of sequential intravenous bolus injections of 150–200-μg sodium nitroprusside (SNP), followed 60 s later by 150–200-μg phenylephrine hydrochloride (PE). All subjects underwent at least two valid test trials. Modified Oxford test trials were separated by at least 15 min to ensure adequate stabilization of BP. Trials were considered valid if the resulting drop and rise in BP was >15 mmHg compared to their baseline values.

For both spontaneous and pharmacological assessments, sympathetic BRS was calculated as the relationship between average integrated MSNA and DBP (Halliwill, 2000). DBP was used due to being more closely related to changes in MSNA than systolic blood pressure (SBP; Sundlof and Wallin, 1978). The nerve trace was time shifted for each individual to take into account inter-individual differences in burst latency. The mean shift applied was 1.35 ± 0.08 s. MSNA recordings were normalized by allocating an amplitude of 1,000 arbitrary units (AU) to the largest baseline burst and calibrating all subsequent nerve activity to the same scale. Burst amplitude was used for the normalization procedure because this is the factor that is affected by electrode placement within the nerve. For the modified Oxford trials, nerve activity for SNP and PE was pooled and averaged over 3 mmHg pressure bins, removing any potential influence from non-baroreflex stimuli (Ebert and Cowley, 1992; Tzeng et al., 2009). Any DBP bins not containing cardiac cycles were excluded. The normalized amplitude and burst duration, in seconds, were incorporated into the calculation for integrated area. The BRS slope was calculated from the relationship between the integrated area of each bin's signal average and DBP. A weighting was applied according to the number of cardiac cycles per bin, which ensures that bins containing the fewest cardiac cycles (generally the highest and lowest DBP bins) had a lower weighting than those containing more cardiac cycles (Taylor et al., 2015). The acceptance level of arterial baroreflex slopes was set at *r* ≥ 0.5 (Taylor et al., 2015). For the modified Oxford method each subject's BRS was presented as the average slope of the two trials.

Cardiac BRS was also calculated using both spontaneous recordings (sequence method) and the modified Oxford method. The same modified Oxford trials described above were used to compute cardiac BRS by plotting R–R interval against SBP with 3 mmHg bins and an acceptance level of *r* ≥ 0.5. The SBP values were matched to the corresponding heartbeat for R–R intervals >800 ms or a one beat delay for shorter heartbeat <800 ms in order to account for arterial baroreflex delays (Eckberg and Eckberg, 1982; Atkinson et al., 2011). For the sequence method, “down” and “up” sequences were identified. Falls in SBP and R–R interval of three or more consecutive cardiac cycles were defined as down sequences. Increases in SBP and R–R interval of three or more consecutive cardiac cycles were defined as up sequences. Changes in systolic BP had to meet a threshold of 1 mmHg and changes in R–R interval a threshold of 6 ms (Parati et al., 1988). For each of the sequences, cardiac BRS was quantified by plotting R–R interval against SBP, with an acceptance level of *r* ≥ 0.8 (Taylor et al., 2015). Mean values for up and down sequences combined were calculated for each subject.

### Spectral and transfer function analysis

Transfer-function analysis of steady-state baseline data was used to quantify dynamic CA (Zhang et al., 1998; Tzeng et al., 2010). Beat-to-beat mean arterial pressure (MAP) and MCAv_mean_ signals were spline interpolated and resampled at 4-Hz for spectral and transfer function analyses based on the Welch algorithm. Initially, each recording was divided into five windows each overlapping by 50%, after which the data were linearly detrended and passed through a Hanning window before fast Fourier transform analysis. For TFA, the cross-spectrum between MAP and MCAv_mean_ was determined and divided by the MAP autospectrum. This approach provides measures of spontaneous MAP and MCAv_mean_ spectral power and mean values of transfer function coherence, gain, and phase in the very-low (0.02–0.07 Hz), low- (0.07–0.20 Hz, and high-frequency (0.20–0.30 Hz) ranges (Zhang et al., 1998). However, for the purposes of this study only LF transfer function gain was used in subsequent statistical analysis since this variable provides an estimate of cerebral autoregulation. The low-frequency range is used as it is the only frequency range not associated with fluctuations caused by respiratory frequency. The low frequency range has also been shown to most precisely define changes caused by autoregulatory mechanisms (Diehl et al., 1995; Zhang et al., 1998). In this range, impairment of CBF regulation is represented by an elevation of transfer function gain (Zhang et al., 2002).

### Tiecks autoregulatory index

Tiecks ARI (Tiecks et al., 1995) was used to separately quantify dynamic CA from the transient hypotension caused by the release of the bilateral thigh cuffs. This technique involves the inflation of cuffs around the thighs (Hokanson, Bellevue, WA) to suprasystolic levels (220 mmHg) for 3 min, followed by rapid deflation. ARI is derived by fitting a 30 s window of MAP and CBFv post-hypotensive stimulus (i.e., upon thigh cuff deflation) to a mathematical model incorporating both gain and latency. From the model, 10 template models of CBFv response based on the post-hypotensive MAP are produced, representing each integer from 0 to 9, with 0 representing no cerebral buffering capacity and 9 representing a strong cerebral buffering capacity. The index of the model curve best fitting the actual CBFv response is the subject's ARI-value. To do this, the following second-order differential equation is applied:

$$\begin{array}{l} {\text{dP}}_{\text{n}}=\text{MAP}-{\text{MAP}}_{\text{base}}/{\text{MAP}}_{\text{base}}-\text{CCP}\\ \text{ x}2_{\text{n}}=\text{x}2_{\text{n}}+\left(\text{x}1\text{n}-2\text{D x}2_{\text{n}-\text{1}}\right)/{\text{f}}^{*}\text{T}\\ \text{ x}1_{\text{n}}=\text{x}1_{\text{n}-\text{1}}+\left({\text{dP}}_{\text{n}}-\text{x}2_{\text{n}-\text{1}}\right)/{\text{f}}^{*}\text{T}\\ {\text{ mV}}_{\text{n}}={{\text{MCAV}}_{\text{base}}}^{*}\left(1+{\text{dP}}_{\text{n}}-{\text{k}}^{*}\text{x}2_{\text{n}}\right) \end{array}$$

where dPn is the normalized change in mean arterial pressure (MAP) relative to the control value (MAPbase). This is adjusted for an estimated critical closing pressure (CCP) of 12 mmHg. x2n and x1n are state variables that are equal to 0 at baseline. mVn is modeled mean velocity, MCAvbase is baseline MCAvmean, f is the sampling frequency (1,000 Hz) and *n* is the sample number. Ten combinations of parameters T (time constant), D (dampening factor), and k (autoregulatory gain) are predetermined, each representing a different level of cerebral buffering capacity, and the one with the best fit represents the individual's ARI.

### Blood pressure variability

BP variability was defined as the SDs of averaged beat-to-beat SBP, MAP, and DBP during the 5-min supine baseline recording. The corresponding coefficients of variation were derived as the SD, divided by the mean and multiplied by 100.

### Statistics

Relationships between sympathetic BRS and CA were described using Pearson's product moment correlation coefficients and least squares regression. Values were expressed as mean ± *SD* unless otherwise stated. Statistical significance was defined a priori at *p* < 0.05.

## Results

### Baseline

Of the 14 subjects that participated, nerve recordings were successfully obtained from 11 subjects based on criteria mentioned in the methods. The mean ± *SD* physiological data are presented in Table 1, including resting MSNA burst frequency and incidence.

**Table 1:** Baseline characteristics.

**Variable**	**Mean ±*SD***
Age, year	24 ± 3
Weight, kg	89 ± 11
Height, m	1.8 ± 0.06
Body mass index, kg/m^2^	27.3 ± 4.0
Heart rate, beats/min	60 ± 15
Systolic Blood Pressure, mmHg	126 ± 9
Diastolic Blood Pressure, mmHg	76 ± 9
ETCO_2_, mmHg	40.8 ± 2.2
Breathing frequency, Hz	0.3 ± 0.04^*^
MSNA burst frequency, bursts/min	22 ± 8
MSNA burst incidence, bursts/100 heartbeats	39 ± 18
MSNA burst latency, s	1.35 ± 0.08

*MSNA, Muscle sympathetic nerve activity. Values are reported as mean ± SD*.

* *None of the subjects breathed at a frequency below 0.2Hz*.

**Figure 1:**
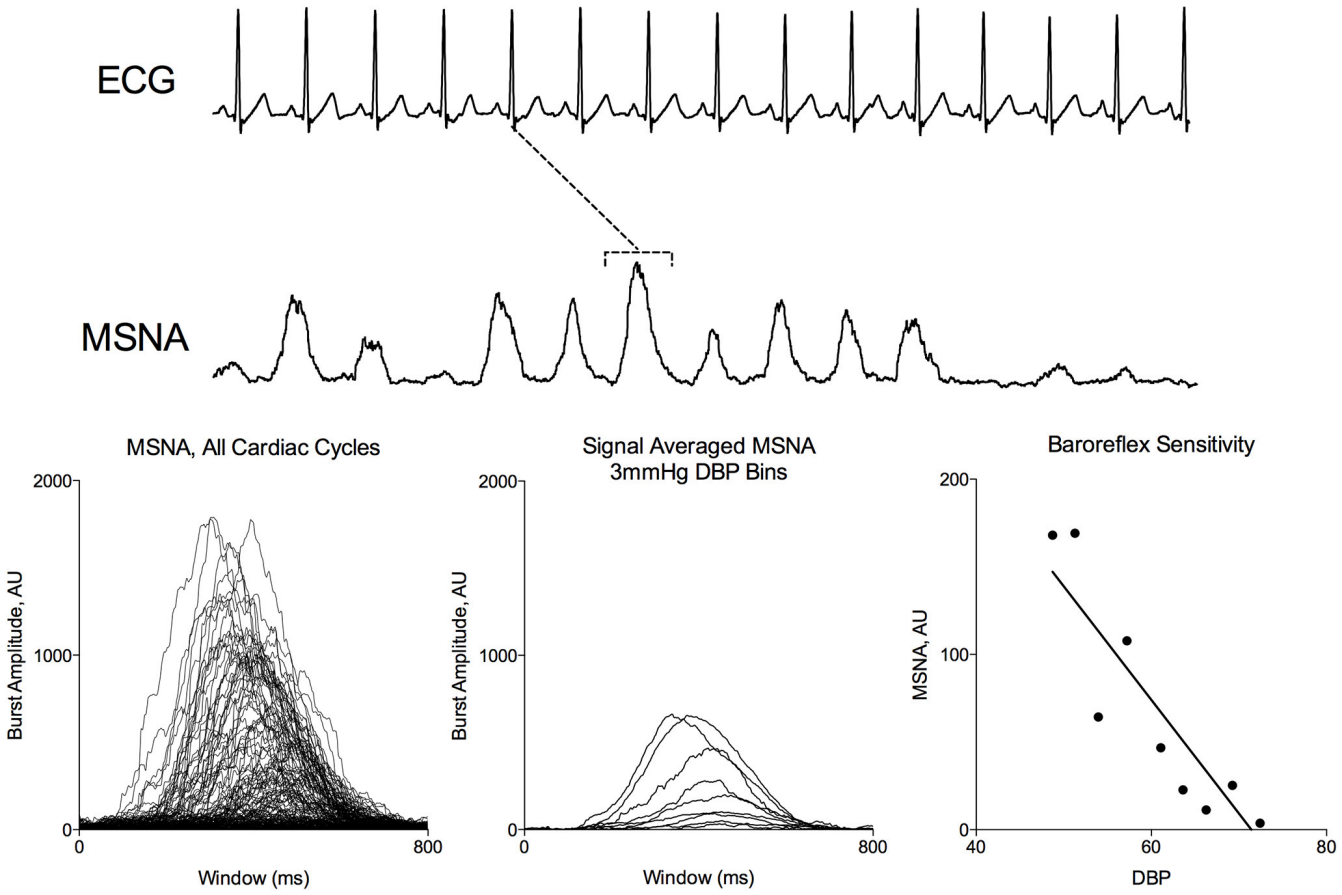
Signal Averaged Analysis of MSNA during Modified Oxford. MSNA activity corresponding to each ECG R peak (dotted line) were binned according to DBP. Average integrated MSNA was plotted for each 3 mmHg pressure bin.

Figure 1 demonstrates segregated signal averaging in a representative subject during a modified Oxford trial. MSNA signals associated with each cardiac cycle are segregated into 3-mmHg DBP bins and the signals for each bin are averaged. BRS was quantified as the slope of the line of best fit of the relationship between DBP and average MSNA integration.

### Arterial baroreflex sensitivity and cerebral autoregulation

**Figure 2:**
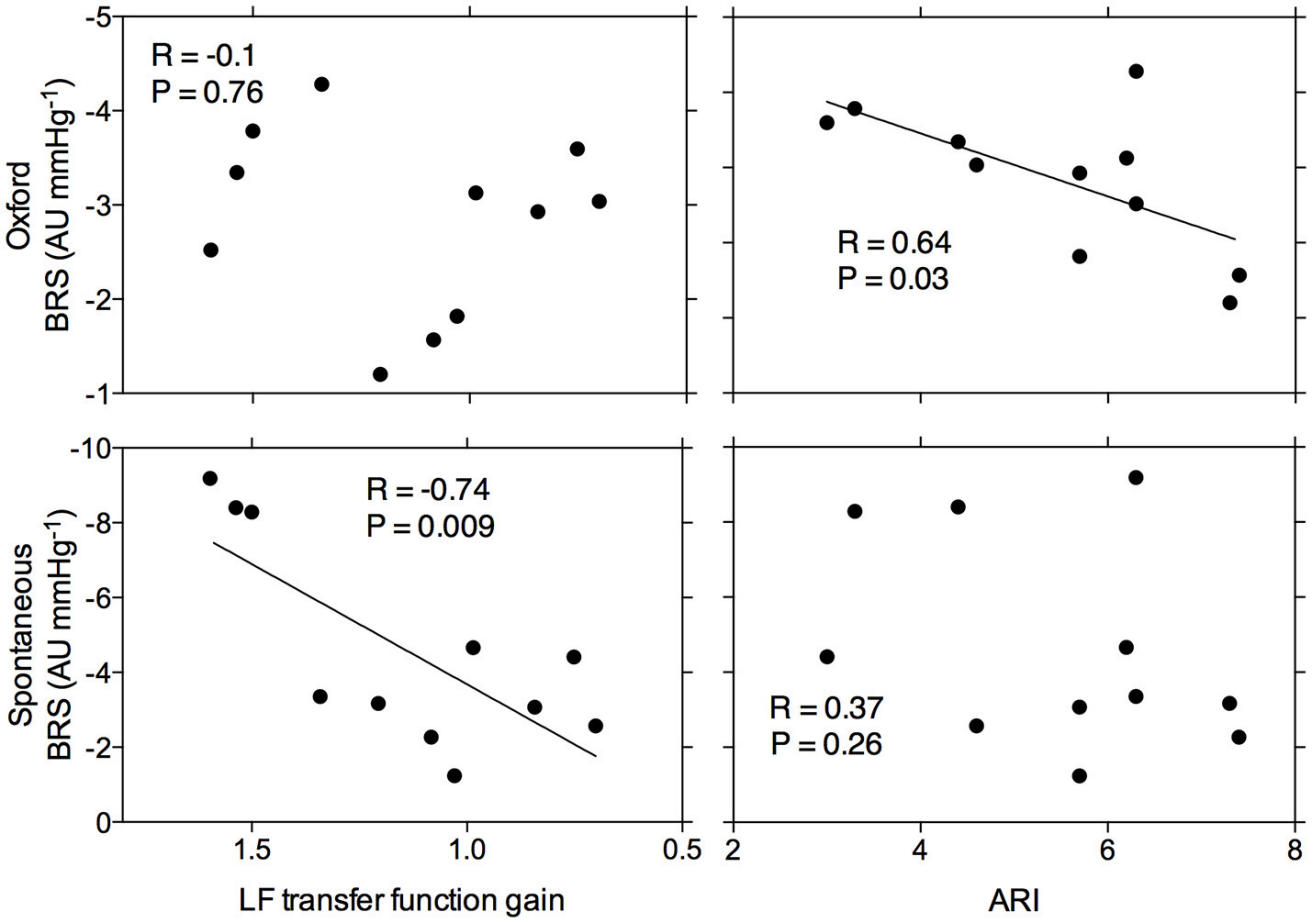
Relationship between arterial baroreflex sensitivity as assessed by the modified Oxford method and spontaneously and cerebral autoregulation as assessed by Tiecks' Autoregulatory Index and transfer function analysis gain. (LF = low frequency). Each point represents data for a given individual.

Figure 2 summarizes the relationship between ARI and TFA gain, and BRS, as quantified through spontaneous and modified Oxford recordings. The data demonstrate that ARI was significantly related to BRS (*r* = 0.64, *p* = 0.03) when using the modified Oxford method but no significant relationship was found with spontaneous BRS (*r* = 0.37, *p* = 0.26). Conversely, TFA gain was not related to BRS using the modified Oxford method (*r* = −0.1, *p* = 0.76) but there was a significant relationship to spontaneous BRS (*r* = −0.74, *p* = 0.009). A more negative value for sympathetic BRS indicates more effective arterial baroreflex regulation, and a lower transfer function gain reflects greater cerebral autoregulation. Therefore, these outcomes indicate that males with attenuated CA have greater sympathetic BRS (and vice versa). Relationships between cardiac BRS and CA did not reach significance regardless of the method used (*p* > 0.05).

### Blood pressure variability

Table 2 summarizes the relationship between CA and BRS to indexes of BPV. BPV and BP variation coefficients were not related to modified Oxford BRS or ARI but most were significantly correlated to spontaneous BRS and TFA gain. Because a greater BRS is represented by a more negative value, the significant positive R-values for the relationship between BPV and BRS are representative of an inverse relationship, meaning that a greater BRS is associated with a lower BPV. Because a smaller TFA gain is associated with a greater CA, the significant negative R-values for the relationship between BPV and TFA gain demonstrate a positive relationship between BPV and CA.

**Table 2:** Relationships between BPV and sympathetic arterial BRS and CA.

	**Oxford BRS**		**Spontaneous BRS**		**ARI**		**TFA gain**	
**Variable**	***R***	***P***	***R***	***P***	***R***	***P***	***R***	***P***
SD SBP, mmHg	−0.20	0.56	0.33	0.32	−0.03	0.93	−0.54	0.09
SD MAP, mmHg	−0.14	0.69	0.60	0.052	−0.20	0.55	−0.68	0.02^*^
SD DBP, mmHg	−0.09	0.78	0.62	0.041^*^	−0.19	0.57	−0.72	0.01^*^
COV SBP, %	0.001	1.0	0.49	0.12	0.0094	0.98	−0.70	0.017^*^
COV MAP, %	0.20	0.56	0.76	0.006^*^	−0.010	0.98	−0.80	0.0032^*^
COV DBP, %	0.22	0.52	0.73	0.01^*^	0.018	0.96	−0.79	0.0036^*^

*SD SBP, Systolic blood pressure standard deviation; SD MAP, mean arterial pressure standard deviation; SD DBP, diastolic blood pressure standard deviation; COV SBP, systolic blood pressure coefficient of variation; COV MAP, mean arterial pressure coefficient of variation; COV DBP, diastolic blood pressure coefficient of variation*.

* Statistically significant, P < 0.05

## Discussion

This study is the first to examine the inter-individual relationships between sympathetic BRS and CA in healthy humans. Consistent with our hypothesis, we found that CA, as assessed by LF transfer function gain and ARI, is inversely related to spontaneous and pharmacological indexes of sympathetic BRS, respectively. These data indicate that individuals with an attenuated BRS tend to have a greater CA (and vice versa), which suggests that among young normotensive men, a reciprocal balance between sympathetic arterial baroreflex function and CA is important in maintaining stable cerebral perfusion. Additionally, we observed a significant inverse relationship between quantifications of BPV and BRS and a positive relationship between some metrics of BPV and CA.

### Interactions between arterial baroreflex and cerebral autoregulation

The traditional concept of CA proposed by Lassen (1959) suggests that CBF plateaus at a near constant value over a broad range of arterial BP levels (60–150 mmHg). According to this construct, arterial baroreflex regulation of BP plays a role in CBF control only in so far as ensuring that BP is maintained within the effective “autoregulatory range.” However, Lassen's concept of CA was based on point estimates of BP and CBF and it therefore does not fully describe the dynamic relationships between BP and CBF over a broad range of physiologically relevant timescales. The advent of high temporal resolution transcranial Doppler has shown that CA is a frequency-dependent process that achieves relative (not absolute) buffering of CBF against dynamic BP perturbations. Therefore, both arterial baroreflex control of BP and autoregulation of cerebrovascular resistance contribute to CBF regulation. However, precisely how these two processes relate to each other is not well-understood.

In theory, at least three potential relationships are possible. First, there may be no relationship since CA is mediated by a multitude of mechanisms including myogenic and metabolic processes that can occur independently of cerebral sympathetic drive. Second is that individuals with high sympathetic BRS might also be expected to have better CA. This is plausible since the sympathetic nervous system regulates both systemic vascular resistance as well as regional cerebrovascular resistance. The third possibility is that individuals with high sympathetic BRS might have lower CA since there might be fewer BP instabilities for the cerebral circulation to contend with. The current study shows that the latter relationship appears to hold true for young healthy men. The relationship found between spontaneous BRS and TFA gain suggests that individuals with high sympathetic BRS have attenuated CA, and vice versa. These findings are also reflected in the relationship between modified Oxford sympathetic BRS and ARI. There was no correlation between spontaneous BRS and ARI or modified Oxford BRS and TFA gain, although it is likely that the lack of correlation may be related to whether the approach was spontaneous or involved active perturbations in blood pressure. Both TFA gain and spontaneous BRS are derived from spontaneous baseline data, whereas ARI and the modified Oxford BRS are both representative of a response to a driven change in BP. Thus, it appears that correlations existed only when metrics of BRS and CA quantification were derived from a similar BP input.

Because the sympathetic arm of the arterial baroreflex plays a large part in BP regulation (Liu et al., 2002; Sakamoto et al., 2014) and cardiac BRS and sympathetic BRS are not correlated in young males (Serrador et al., 2000; Taylor et al., 2015), an understanding of the sympathetic arterial BRS relationship to CA is critical to understanding the relationship between the arterial baroreflex and CA. This study demonstrates an inverse relationship between the two mechanisms and therefore suggests that the integration between CA and arterial baroreflex function extends to both the cardiac and sympathetic components of the arterial baroreflex. These results are also consistent with previous results with regards to the relationships between BPV and both BRS and CA. It has previously been reported that high BPV is associated with low cardiac BRS (Tzeng et al., 2010) and, although there was not sufficient statistical power to replicate these findings with cardiac BRS, the current study confirms that a similar relationship exists with sympathetic BRS. This is consistent with our understanding of the role of the arterial baroreflex in maintaining blood pressure homeostasis. The current study also confirms previous findings that high BPV is associated with more effective CA (Tzeng et al., 2010), which is further evidence to support the conclusion that CA is more effective when BP is less tightly controlled, i.e. a compensatory relationship.

### Potential explanations and implications

Physiological diversity is a ubiquitous feature of living systems that underpins many phenotypic patterns of adaptation and disease. However, the origins of such diversity are poorly understood with functional variations often dismissed as random measurement noise. Our findings suggest this view is overly simplistic since the inter-individual variance in some functional parameters, such as sympathetic BRS and CA, appear to be underlain by regulatory structure and organization. It is important to emphasize that correlations do not imply causation and therefore it is not possible for us to pinpoint precisely how or why these relationships exist. However, we speculate that consideration of dynamical systems theory and its intersection with evolutionary theory may give insight into the possible origins of these relationships.

From a dynamic systems theory perspective, physiological mechanisms such as the arterial baroreflex and CA are highly complex systems that involve a multitude of components from genes to molecules and cells and higher order assemblies (Hirsch et al., 2004; Jaeger and Monk, 2014). In theory, the constituent components can be combined in innumerable ways yet in reality the configuration, dimensionality and complexity of physiological systems can be reduced to certain preferred states of co-ordination or “attractor” states (Strogatz, 2000; Hirsch et al., 2004). The defining feature of an attractor state is that system dynamics are ordered, stable, and optimally configured to support the functions of the system (Jaeger and Monk, 2014). Thus, the apparent “functional equilibria” that exists between sympathetic BRS and CA could reflect, at least in part, the nature of attractor states that defines how the systems optimally converges to regulate CBF among young healthy males. However, it must be acknowledged that time is an important dimension in dynamic systems theory and in this study measurements were made only at a single time point. Therefore, it is possible that differentiation of attractor states could alter these relationships as people age and continue to physiologically interact with the environment.

Another intriguing dimension to these concepts is that the precise nature of dynamic systems may potentially have evolutionary origins and implications. One of the central tenants of the modern evolutionary synthesis is that natural selection acts on genes or individuals (Noble et al., 2014). Physiological function is generally not considered important except in constraining the fitness of individual organisms in terms of its reproductive success after genetic mutations have created the possibility of an advantage (Wagner, 1984; Maynard-Smith, 1985). However, selection may also occur at a population level. The presence of physiological variation, such as that observed here in CBF maintenance, could prove advantageous for overall population survival.

### Methodological considerations

Several limitations must be considered in context of these results. This study focuses primarily on young healthy males and the findings from this study can therefore only be generalized to this population. Recent studies have demonstrated age and gender related variances in vascular transduction of MSNA (Hart et al., 2009a,b). Variances in neural transduction would alter the effectiveness of the arterial baroreflex and may affect the relationship between the arterial baroreflex and CA. Further investigation is warranted on the relationship between the arterial baroreflex and CA in female and older male populations. The correlations in this study varied depending on the method of quantifying CA. We have previously demonstrated a lack of convergent validity between quantification methods of CA (Tzeng et al., 2012) and the physiological interpretation of each method's results warrants further research. While there is a lack of convergence between CA quantification methods, our results derived from Tiecks ARI and TFA gain both support the concept of a compensatory relationship between CA and the arterial baroreflex. Whilst the current findings suggest that such a relationship exists, the study design does not allow for this relationship to be challenged. It is therefore not known whether there is a dynamic element to this relationship within the individual, such that if one regulatory mechanism is compromised the other is enhanced. Further research is needed in order to challenge the compensatory relationship between BRS and CA.

#### Velocity vs. flow

The use of transcranial Doppler derived measurements of MCAv as a representative of CBF is dependent on the assumption that MCA diameter stays constant. Due to the quadratic influence of vessel cross sectional area on diameter and consequently flow, minor changes in vessel constriction would result in substantial disparities between changes in MCAv and CBF. However, previous studies have demonstrated no change in MCA diameter in resting and lower body negative pressure conditions (Serrador et al., 2000). With a constant diameter, we can assume that changes in MCAv accurately represent changes in CBF.

#### Assessment of cerebral autoregulation

For this study, we assessed the buffering capacity of the cerebral vasculature through both TFA of spontaneous fluctuations in MAP and CBFv at baseline and Tiecks ARI during thigh cuff deflation. A drawback to both of these techniques is that they both assume linearity between BP and CBF and do not account for the non-linear properties associated with CA (Aaslid et al., 2007; Panerai, 2009; Tzeng et al., 2012). Furthermore, the thigh cuff technique is limited by the fact that only responses to falling pressures are examined, thus potentially missing any hysteresis that may exist.

#### Assessment of sympathetic arterial baroreflex

Currently there is no gold standard technique for the quantification of BRS. While assessment of BRS under spontaneous resting conditions is common, external influences on nerve activity cannot be ruled out and only a narrow range of associated BP-values are represented. Pharmacological BP manipulations may have unknown external effects on the arterial baroreflex but the use of vasoactive drugs allows the quantification of nerve activity responding to all BP-values ranging from hypo- to hypertensive. This results in a slope derived from a greater number of points making it a more robust assessment of BRS. In terms of analysis, the integrated signal averaging approach developed by Halliwill (2000) was employed because it takes into account both MSNA burst incidence and MSNA burst strength. However, in light of evidence suggesting that these two components may be modulated by two different sites (Kienbaum et al., 2001), this could also be seen as a disadvantage. With the integrated approach it is not possible to discern any separate relationships pertaining to burst incidence or burst strength alone.

#### Neural transduction

MSNA is used with the assumption that it plays a direct role in regulating peripheral arterial resistance. MSNA is not a direct measure of peripheral resistance in itself but is positively related to total peripheral resistance in young males (Charkoudian et al., 2005). Though a good indicator of vasoconstrictor tone in young males, this relationship may be altered or nonexistent in older male, young female and older female populations (Franklin et al., 1997; Charkoudian et al., 2006; Hart et al., 2009a,b). Therefore, the effects of age and gender need to be considered before assuming these results provide a collective representation for all humans.

## Conclusions

In summary, the findings suggest that CA is inversely related to sympathetic BRS as quantified using the modified Oxford method and spontaneous techniques. This is consistent with previous findings regarding the relationship between cardiac BRS and CA, suggesting that the relationship is representative of the arterial baroreflex in its entirety.

## Author contributions

Experiments were performed at the Centre for Translational Physiology at the University of Otago Wellington. All authors were involved in the design of the experiments and/or data acquisition and analysis of the data, as well as the writing or editing of this manuscript. All authors approved the final version of the manuscript and agree to be accountable for all aspects of the work.

### Conflict of interest statement

The authors declare that the research was conducted in the absence of any commercial or financial relationships that could be construed as a potential conflict of interest.
